# Iron deficiency anemia as a risk factor for pulp disease in children from the central Peruvian jungle: a case‒control study

**DOI:** 10.1590/1678-7757-2024-0014

**Published:** 2024-06-14

**Authors:** Jhair Alexander LEON-RODRIGUEZ, María ESPINOZA-SALCEDO, Yovana Melisza GUTIERREZ-POLANCO, Jherson David LEON-RODRIGUEZ, Araceli Antonella LOPEZ-TISNADO, Omaly Sulay RIVERA-CRUZ

**Affiliations:** 1 Antenor Orrego Private University Postgraduate School Trujillo Peru Antenor Orrego Private University, Postgraduate School, Trujillo, Peru.; 2 San Martin National University Postgraduate School Tarapoto Peru San Martin National University, Postgraduate School, Tarapoto, Peru.; 3 Los Angeles Catholic University of Chimbote Dental School Trujillo Peru Los Angeles Catholic University of Chimbote, Dental School, Trujillo, Peru.; 4 National university of Trujillo Postgraduate School Trujillo Peru National university of Trujillo, Postgraduate School, Trujillo, Peru.

**Keywords:** Anemia, Case-control studies, Iron deficiency, Pulp

## Abstract

**Aim:**

To investigate iron-deficiency anemia as a risk factor for dental pulp disease in children from the central Peruvian jungle.

**Methodology:**

A case-control study was carried out with 270 children, of which 90 referred to cases and 180, to controls. Patients with pulp disease were diagnosed according to the criteria of the Association of Endodontists and the American Board of Endodontics. A specific questionnaire was used to assess ferrous sulfate consumption, maternal education level, maternal age, occupation, and household income. Data were analyzed using Pearson’s correlation coefficient and a binary logistic regression.

**Results:**

Iron deficiency anemia offers a risk factor for pulp disease in children (OR 7.44, IC 95% 4.0-13.8). According to multivariate analysis using binary logistic regression, ferrous sulfate consumption (OR 13.8, IC 95% 5.6.33.9), maternal education level (OR 2.4, IC 95% 1.1-5.3), maternal age (OR 7.5, IC 95% 2.9-19.4), household income (OR 4.0, IC 95% 1.6-9.6), and caries (OR 10.7, IC 95% 4.5-25.7) configured independent factors that were statistically associated with pulp disease.

**Conclusion:**

Iron deficiency anemia, ferrous sulfate consumption, maternal education level, maternal age, household income, and dental caries were positively associated with pulp disease in children

## Introduction

The human body requires a balance between physiological, nutritional, and environmental factors to maintain its optimal functioning.^1^ An imbalance due to iron deficiency leads to the development of iron deficiency anemia, which has a series of adverse consequences that negatively impact the body.^2^ Anemia represents a serious public health problem, affecting 20% of children worldwide and 38.6% of children in Peru.^3^

Iron deficiency, characteristic of iron deficiency anemia, often leads to the prescription of ferrous sulfate as the primary treatment in the form of syrups and drops.^4^ Iron salts react with gingival crevicular fluid and subgingival bacterial metabolites, staining teeth.^5^ Iron supplements typically have a highly acidic pH (usually from 1 two 5), compromising dental enamel microhardness and increasing the risk of cavities. However, the severity of the damage varies depending on supplement acidity, frequency, duration, and method of administration.^6^

The oral cavity is composed of both soft and hard structures, including teeth, which consist of enamel, dentin, and pulp, tissues that contain iron.^7^ Affecting these structures can initiate a dysbiosis that leads to dental caries.^8^

Severe carious lesions in children are associated with low levels of serum ferritin (<12 µg/L) ^9^ and hemoglobin (<11.5 g/dL).^10^ Iron deficiency reduces the buffering capacity of saliva, depriving it of its ability to counteract pH changes and protect oral tissues against acids from food or dental plaque. Therefore, it increases cariogenic potential.^11^ The progression of carious lesions facilitates bacterial invasion toward the dental pulp. The exposure of the pulp to pathogens triggers a localized inflammatory response that releases inflammatory mediators. This process shows an increase in pulp pressure, resulting in acute pain in pediatric patients, a characteristic symptom of pulpal disease.^12^

It is important to recognize that prolonged consumption of ferrous sulfate may be associated with dental stains. This situation, combined with anemia, may predispose children to a higher risk of developing dental caries, potentially contributing to the onset of pulp diseases.^13^

According to the classification system developed by the American Association of Endodontics, the diagnosis of pulpal and periapical diseases should be based on clinical and radiographic findings.^14^ The diagnosis of reversible pulpitis is based on objective criteria and subjective tests, reducing inflammation and restoring normal pulp function after appropriate treatment.^15^ Irreversible pulpitis may be symptomatic or asymptomatic and is characterized by findings that indicate the need for root canal treatment as the inflamed vital pulp is unable to heal on its own.^16^ Pulpal necrosis is a clinical diagnostic feature indicating the death of the dental pulp.^17^

The study was carried in the central Peruvian jungle, in the district of Pichanaki, belonging to the province of Chanchamayo, Department of Junín. This municipality is located at an altitude of 525 meters and has a population of 69,817 inhabitants, of which 9,786 are children aged from 0 to 5 years and 10,099, from 5 to 11 years. The poverty rate in the area totals 56.8%. In 2023, the Primary Care Center of the Social Health Insurance of the Region cared for 4,535 children,^18^ and this was the location from which data were collected.

The theoretical importance of this study lies in its contribution to knowledge by identifying anemia as a risk factor for the onset of pulpal diseases in children from a region in the central jungle of Peru. Anemia constitutes a public health problem that affects children treated in various health establishments of the Ministry of Health and the Peruvian social security system. The methodological relevance of the study is related to the application of validated and greatly reliable instruments that can be used in future research and applied in association with other variables.

Its social importance lies in identifying this disease, which was yet to be investigated in the region and could be affecting the oral health of children, impacting their educational and social performance and affecting their learning process as pulp pathologies could exacerbate this problem.

Therefore, this study aimed to investigate the association between iron deficiency anemia and pulp disease in children from the central Peruvian jungle.

## Methodology

### Study design and ethical approval

This case-control study was designed to analyze anemia as a risk factor for pulp diseases in the central Peruvian jungle. This study fully adhered to the 22 items of the STROBE guideline checklist, the ethical criteria outlined in the Declaration of Helsinki, and the international guidelines for health research. Moreover, informed consent and anonymity were ensured. The project was approved by the Antenor Orrego Private University Bioethics Committee (Resolution No. 0762-2023-UPAO).

### Sampling and sample size

The population consisted of 852 children who received care at the dentistry service of the Pichanaki Primary Care Center of the Peruvian social health insurance. As this was a logistic regression study, the classic Freeman formula was used to calculate the sample size; totaling 90 cases and 180 controls. A simple random probabilistic sampling method was used.

### Subject population

This study included 270 children who received dental treatment at the Pichanaki Primary Care Center of the Social Health Insurance of Peru. The children were divided into two groups. The study group (n=90) included children who were diagnosed with pulp disease and were compared with the control group (n=180), which included healthy children.

### Inclusion and exclusion criteria

For the case group, the inclusion criteria referred to ages from 5 to 11 years, the presence of pulp disease, and previous screening for anemia. Children whose parents requested their voluntary withdrawal from this research, those with dentoalveolar trauma, systemic diseases, psychological alterations, behavioral problems, or some hematological alterations were excluded.

For the control group, inclusion criteria referred to no pulp disease and previous screening for anemia. The exclusion criteria were the same for all the cases. To meet the homogeneity criteria, the controls were taken from the same place as cases. To meet simultaneity criteria, controls were evaluated at the same time as cases. Matching was used to adequately control for variables.

### Procedure and technique

Intra and inter-examiner calibrations were performed under the direction of a registered dental surgeon specialized in pediatric dentistry. The Cohen’s kappa coefficient was evaluated to diagnose pulp disease and dental caries. The result for pulp disease was k=1.00 and, for dental caries, k=0.95, indicating an almost perfect agreement.

The children treated at the Pichanaki Primary Care Center dental service in the Pichanaki district, province of Chanchamayo, in the central Peruvian jungle from May to October 2023 were identified. Children who showed pulp disease were considered as cases and those who showed none, as controls.

During anemia screening, patients with hemoglobin levels greater than or equal to 11.5 g/dL were considered non-anemic, whereas those with hemoglobin levels below 11.5 g/dL were considered anemic. No adjustment of values for altitude was required because the city lies 525 meters above sea level.^10^ To obtain the data, their medical history was reviewed and their last hemoglobin screening performed using the spectrophotometry method was verified.

Cases and controls were selected according to the inclusion and exclusion criteria described above. Complete sociodemographic factor data were recorded (ferrous sulfate consumption, maternal education level, maternal age, occupation, and household income). Additionally, height and weight data were collected to determine malnutrition.

To evaluate pulp disease, the children were clinically evaluated using an odontogram standardized by the Ministry of Health of Peru after the obtention of informed consent from their parents.^19^ The clinical and radiographic aspects, for obtaining a correct diagnosis, were described, and the diagnostic criteria that have been approved by the Association of Endodontists and the American Board of Endodontics^20^ were followed.

To determine the presence of dental caries, data were recorded on an odontogram that has been validated by the Ministry of Health.^19^The dmft index (decayed, missing, and filled primary teeth) and DMFT (decayed, missing, and filled permanent teeth) index^21^ were used to determine the prevalence of caries. The results of this evaluation were subsequently placed in an Excel database for subsequent statistical analysis.

### Statistical analysis

To process the information, the statistical package SPSS, version 27, was used. For descriptive statistics, absolute and relative frequencies were used to generate double-entry frequency tables. For inferential statistics, a bivariate analysis was conducted using the Pearson’s chi-square test. A binary logistic regression was used for the multivariate analysis, with OR and 95% confidence intervals.

## Results

Univariate analysis of the intervening variables showed that ferrous sulfate consumption (p=0.000, OR 17.7, 95% CI 8.8-35.9), maternal education level (p=0.000, OR 3.8, 95% CI 2.2- 6.5), maternal age (p=0.000, OR 6.4, 95% CI 6.4-11.9), household income (p=0.000, OR 3.6, 95% CI 2.2-6.2), and infant dental caries (p=0.000, OR 6.5, 95% CI 3.7-11.7) were significantly associated with pulp disease in infants. However, this study found no significant associations between maternal occupation or chronic malnutrition and acute malnutrition in this population (Table 1).

**Table 1:** Sociodemographic characteristics of pediatric patients aged from 5 to 11 years from the central Peruvian jungle.

Characteristics	Cases n=90	Controls n=180	OR (IC 95%)	p
**Ferrous sulfate consumption**				
Yes	79 (88%)	52 (29%)	OR: 17.7	0.000*
No	11 (12%)	128 (71%)	(IC 95% 8.8-35.9)	
**Maternal education level**				
Professional	50 (56%)	45 (25%)	OR: 3.8	0.000*
Nonprofessional	40 (44%)	135 (75%)	(IC 95% 2.2-6.5)	
**Maternal age**				
≤ 25 years	40 (44%)	20 (11%)	OR: 6.4	0.000*
> 25 years	50 (56%)	160 (89%)	(IC 95% 6.4-11.9)	
**Occupation**				
Housewife	23 (26%)	63 (35%)	OR: 0.7	0.116
Formal work	67 (74%)	117 (65%)	(IC 95% 0.4-1.1)	
**Household income**				
Low	54 (60%)	53 (29%)	OR: 3.6	0.000*
High	36 (40%)	127 (71%)	(IC 95% 2.2-6.2)	
**Chronic malnutrition**				
Yes	14 (16%)	23 (13%)	OR: 1.3	0.532
No	76 (84%)	157 (87%)	(IC 95% 0.6-2.9)	
**Acute malnutrition**				
Yes	6 (7%)	8 (4%)	OR: 1.6	0.438
No	84 (93%)	172 (96%)	(IC 95% 0.6-4.6)	
**Dental caries**				
Yes	70 (78%)	63 (35%)	OR: 6.5	0.000*
No	20 (22%)	117 (65%)	(IC 95% 3.7-11.7)	

*p value < 0.05: Statistically significant

According to the bivariate analysis between anemia and the presence of pulp disease, the frequency of anemia totaled 82% in patients with pulp disease and 38% in patients without pulp disease. The chi-squared test (p=0.000) showed a significant risk effect, with an odds ratio of 7.44 and a 95% confidence interval: (4.0 – 13.8), which enabled us to claim that infants with anemia had a greater probability of developing pulp disease (Table 2).

**Table 2:** Association between anemia and pulp disease in pediatric patients aged from 5 to 11 years from the central Peruvian jungle.

Anemia	Pulp disease		Total	X^**2**^	OR
	Yes	No			
Yes	74 (82%)	69 (38%)	143	44.648	7.4
No	16 (18%)	111 (62%)	127	p=0.000	IC 95% 4.0-13.8
Total	90 (100%)	180 (100%)	270		

According to the multivariate analysis using binary logistic regression, ferrous sulfate consumption, maternal education level, maternal age, household income, and presence of caries infants configured significant independent factors that were statistically associated with pulp disease: (p=0.000, OR 13.8, 95% CI 5.6 – 33.9), (p=0.023, OR 2.4, 95% CI 1.1 – 5.3), (p=0.000, OR 7.5, 95% CI 2.9 – 19.4), (p=0.002, OR 4.0, 95% CI 1.6 – 9.6), and (p=0.000, OR 10.7, 95% CI 4.5 – 25.7), respectively. However, occupation or chronic malnutrition and acute malnutrition showed no significant associations (Table 3).

**Table 3:** Multivariate analysis of the intervening factors in pediatric patients aged from 5 to 11 years from the central Peruvian jungle.

Variable	Statisticians				p
	OR	IC 95%	Wald	Coefficient B	
Ferrous sulfate consumption	13.8	5.6 – 33.9	32.87	2.63	0.000*
Maternal education level	2.4	1.1 – 5.3	5.18	0.89	0.023*
Maternal age	7.5	2.9 – 19.4	16.89	2.01	0.000*
Occupation	0.7	0.3 – 1.7	0.54	-0.33	0.461
Household income	4	1.6 – 9.6	9.79	1.39	0.002*
Chronic malnutrition	1.6	0.6 – 4.4	0.71	0.45	0.398
Acute malnutrition	1.7	0.4 – 7.6	0.52	0.54	0.476
Dental caries	10.7	4.5 – 25.7	28.16	2.37	0.000*

*p value < 0.05: Statistically significant.

## Discussion

Anemia, a highly prevalent problem worldwide, mainly affects the pediatric population.^2^ According to the Peruvian Ministry of Development and Social Inclusion, 42.9% of children aged from six to 35 months suffer from anemia in the central Peruvian jungle,^22^ which remains a serious public health problem. Pulp disease causes pediatric patients to see dental consultations more frequently.^23^ These patients represent from 12 to 13% of all outpatient visits, according to reports from the Ministry of Health.^24^

This investigation determined the influence of the sociodemographic characteristics of pediatric patients on the occurrence of pulpal disease by univariate and multivariate analyses. It showed significant associations between infants with pulpal disease and ferrous sulfate consumption, maternal education level, maternal age, household income, and dental caries.

These findings align with research conducted by Balcázar, et al.^23^ (2017) in Mexico and by Estrela, et al.^25^ (2011) in Brazil, who found an association between dental caries and pulp disease. This is attributed to the fact that dental caries involves colonization of the dental surface by acidogenic microorganisms, which demineralize the enamel and facilitates the bacterial invasion of the dental pulp as the carious lesion progresses.

When the dental pulp is exposed to pathogens, a localized inflammatory response occurs, which releases inflammatory mediators. This process shows increased pulp pressure, which results in acute pain in pediatric patients and is a characteristic symptom of pulp disease.^12^

Regarding the association between ferrous sulfate and pulp disease, findings disagree with those of Cameron and Widmer^26^ (2013), who show that ferrous sulfate reacts with the pulp tissue, forming a superficial protective layer called the iron-protein complex. However, ferrous sulfate undoubtedly produces dental pigmentation, as per Pani, et al.^7^ (2015) and Castro^27^ (2021), who explained that the contact of ferrous sulfate with the oral microbiota generates a chemical reaction that results in iron sulfide, which is deposited on the enamel surface, in some cases penetrating it and causing color changes.

Asgari, et al.^28^ (2020) supports the findings of this investigation, which reported that insufficient iron administration can lead to the development of dental caries because ferrous sulfate drops in a cariogenic diet decrease enamel microhardness. Therefore, we can hypothesize that when children consume ferrous sulfate, they can experience the phenomenon of dental pigmentation, which may be related to the appearance and development of cavities, and eventually trigger pulp problems.

Findings on monthly household income agree with Kato, et al.^29^ (2017). The authors found that a low household income category in Japan was strongly associated with a high prevalence of dental caries leading to pulp disease. Low-income households face barriers to accessing regular dental services, education, information on oral hygiene practices, and oral care products such as toothbrushes, toothpastes, and rinses.

This study differs from Balcázar, et al.^23^ (2017), who found no correlation between education and pulp disease. However, we must keep in mind that in Pichanaki, in which this research was carried out, according to data from National Institute of Statistics and Informatics, 78.7% of people older than 25 years of age have no higher education and 96.5% of women have no job,^18^ which enables us to infer that, under a low level of education and a very high poverty rate, the educational level on oral health is usually deficient. Lack of knowledge and poor oral hygiene practices configure risk factors for developing pulp disease.

No scientific evidence supports an association between iron deficiency anemia and pulp disease. However, this study aimed to investigate this association in an area of extreme poverty with a number of pediatric patients with anemia. It found that the frequency of anemia in patients with pulp disease totaled 82%, with an odds ratio of 7.44, indicating that anemia configures a risk factor in pediatric patients with pulp disease.

Iron deficiency anemia hinders the transport of oxygen via the hemoglobin to peripheral tissues, explaining this event.^30-32^ This affects tissue perfusion, resulting in lower oxygen saturation values in teeth compared to those obtained in patients’ fingers. Mulyahela, et al.^33^ (2022) confirmed this finding, explaining that the confined nature of the dental pulp makes this tissue more sensitive when under a lack of oxygen in the bloodstream.

Adequate blood supply to the dental pulp indicates pulp vitality. The key factor for this is the presence of oxygen in the tissue. Although the seemingly indirect relationship, De la Rosa, et al.^34^ (2021), Pozzobon, et al.^35^ (2011), and Bargrizan, et al.^36^ (2016) suggest that oximetry can be used to determine the state of the pulp.

Bozyel, et al.^37^ (2021) indicated that hypoxia is generally accompanied by infection and inflammation and that the initial sign of the reaction of periodontal tissues to hypoxia is inflammation. The local inflammation disturbs microcirculation and induces leukocyte infiltration, leading to blood and oxygen supply deficiency. Similarly, Colombo, et al.^38^ (2020) mentioned that hypoxia physiologically causes vascular injuries to the dental pulp, leading to inflammation and permanently reducing the oxygen supply to the pulp tissue.

Pediatric patients with anemia may be particularly susceptible to hypoxia in the pulp tissue due to the complicated transport of oxygen. Inflammation or injury can trigger the development of pulp disease in these sensitive tissues. The findings of this research remain inconclusive as it ignored other factors, such as diet and dental care. However, it is important to consider the potential impact of ferrous sulfate consumption, maternal education level, maternal age, household income, and dental caries on the development or worsening of pulp disease.

## Conclusion

Anemia configures a risk factor for pulpal disease as 82% of patients with pulp disease suffered from it. According to our multivariate analysis, ferrous sulfate consumption, maternal education level, maternal age, household income, and dental caries constitute factors that are statistically associated with pulp disease.
